# Effectiveness of and Factors Associated with Balloon Adhesiolysis in Patients with Lumbar Post-Laminectomy Syndrome: A Retrospective Study

**DOI:** 10.3390/jcm9041144

**Published:** 2020-04-16

**Authors:** Yul Oh, Dong Ah Shin, Dong Joon Kim, Woojong Cho, Taejun Na, Jeong-Gil Leem, Jin-Woo Shin, Doo-Hwan Kim, Kyung-Don Hahm, Seong-Soo Choi

**Affiliations:** 1 Department of Anesthesiology and Pain Medicine, Asan Medical Center, University of Ulsan College of Medicine, Seoul 05505, Korea; dhdbf@hanmail.net (Y.O.); gochokdj@naver.com (D.J.K.); hiskygh4@hanmail.net (W.C.); gsnadae27@naver.com (T.N.); jgleem@amc.seoul.kr (J.-G.L.); sjinwoo@hotmail.com (J.-W.S.); knaaddict@gmail.com (D.-H.K.); 2 Department of Neurosurgery, Spine and Spinal Cord Research Institute, Severance Hospital, Yonsei University College of Medicine, Seoul 03722, Korea; shindongah@me.com

**Keywords:** epidural adhesiolysis, balloon, post-laminectomy syndrome, lumbar

## Abstract

Post-laminectomy syndrome (PLS) is characterized by chronic pain and complex pathological entity after back surgery. An epidural adhesiolysis is considered an effective treatment option for lumbar PLS. In this study, we retrospectively analyzed the outcome and evaluated the predictive factors of combined epidural adhesiolysis and balloon decompression using inflatable balloon catheters in lumbar PLS cases. One hundred and forty-seven subjects were retrospectively assessed and analyzed. The percentages of patients who exhibited treatment response were 32.0%, 24.5%, and 22.4% of the study population at 1, 3, and 6 months, respectively. In multivariate logistic regression analysis, the pain duration was independently associated with the treatment response six months after combined epidural adhesiolysis and balloon decompression (odds ratio = 0.985, 95% confidence interval = 0.971–0.999; *p* = 0.038). In addition, the receiver operating characteristic curve analysis showed that the area under the curve of pain duration after lumbar surgery was 0.680 (95% confidence interval = 0.597–0.754, *p* = 0.002), with an optimal cut-off value of ≤14 months, sensitivity of 51.5%, and specificity of 81.4% Our results suggest that an early intervention using combined epidural adhesiolysis and balloon decompression in lumbar PLS patients may be associated with a favorable outcome, even though it has limited effectiveness.

## 1. Introduction

Recurrent and chronic lower back pain (LBP) with or without sciatica is one of the leading causes of a poor quality of life. To manage LBP and sciatica, investigations are ongoing to evaluate the evidence of superiority between conservative management and surgical treatment [1,2]. A recent study evaluated the clinical importance of spine surgery and showed favorable outcomes within four years rather than five years [1,3]. However, Fritsch et al. found that reintervention rates following lumbar discectomy between 1965 and 1990 ranged from 5% to 33% [4]. Thus, interventional treatment for recurring or persistent symptoms after lumbar spinal surgery is necessary but the outcome is not promising because the symptoms have many subetiologies and are often overlapping [5]. Among many etiologies, epidural adhesion of fibrosis has been suggested as one of the important causative factors of recurrent back and leg pain after lumbar surgery. According to Amirdelfan et al., percutaneous epidural adhesiolysis showed good results in the management of lumbar post-laminectomy syndrome (PLS), when compared with many other treatment options [6,7]. An inflatable balloon catheter has been recently developed to conduct safe and successful epidural adhesiolysis in chronic lumbar radicular and back pain cases. Our previous studies reported its outcomes and associated factors in patients with lumbar spinal stenosis and/or herniated disc disease without a history of lumbar surgery [8,9,10]. Therefore, we speculated that combined epidural adhesiolysis and balloon decompression could be an effective treatment option for lumbar PLS. In the present study, we thus aimed to evaluate the outcomes and determine the associated factors of successful outcome after combined epidural adhesiolysis and balloon decompression in patients with lumbar PLS based on retrospective analysis.

## 2. Materials and Methods

We analyzed retrospectively collected data from patients diagnosed with lumbar PLS and treated with combined epidural adhesiolysis and balloon decompression using an inflatable balloon catheter in a single pain clinic of a tertiary medical center in Seoul, Republic of Korea. All participants gave informed consent and this study protocol was approved by the Ethical Committee of the Asan Medical Center (ethical approval number 2018-1219). This study was conducted in accordance with the Declaration of Helsinki.

### 2.1. Participants

We searched our institution’s Information Technology of Service Management (ITSM) system between January 2014 and March 2018 using the terms “history of lumbar spinal surgery”, “epidural adhesiolysis using an inflatable balloon catheter”, and “chronic low back or leg pain”. Patients were included in the study only if they met the following criteria: (1) at least 20 years of age; (2) had a history of spinal surgery in the lumbar spine confirmed with imaging; (3) chronic back and leg pain with severity of at least 6 points on the numerical rating pain scale (NRS) within three months after spinal surgery [11]; and (4) symptoms were not relieved or had not subsided within one month after previous transforaminal or interlaminar epidural steroid injection combined with exercise, medical treatment, or physiotherapy. We excluded patients who satisfied any of the following conditions: (1) less than three months of pain following spinal surgery; (2) other causes of symptoms such as vascular or other systemic disease; (3) allergy to local anesthetics, contrast dye, or steroids; (4) presence of coagulopathy; (5) pregnant or lactating; (6) presence of a systemic infection or injection site infection; (7) presence of malignancy; and (8) an unstable medical or psychiatric condition. All patients were allowed to receive noninvasive therapies including medication, exercise, and physiotherapy following the procedure.

### 2.2. Procedure: Combined Epidural Adhesiolysis and Balloon Decompression

**Figure 1:**
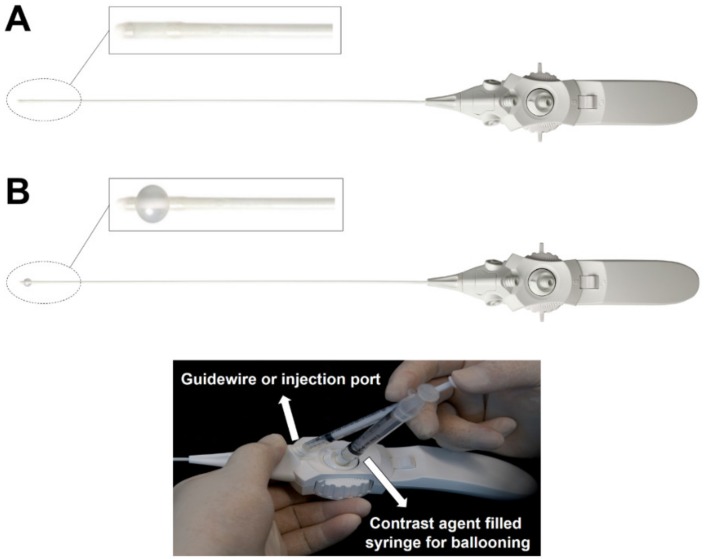
An inflatable balloon catheter (ZiNeu^®^, JUVENUI, Seoul, Korea). An elastic inflatable balloon is attached to the end of the catheter tip. The balloon can be inflated with about 0.13 mL of contrast dye. (**A**) A deflated state of balloon at the tip of the catheter. (**B**) An inflated state of balloon at the tip of the catheter.

**Figure 2:**
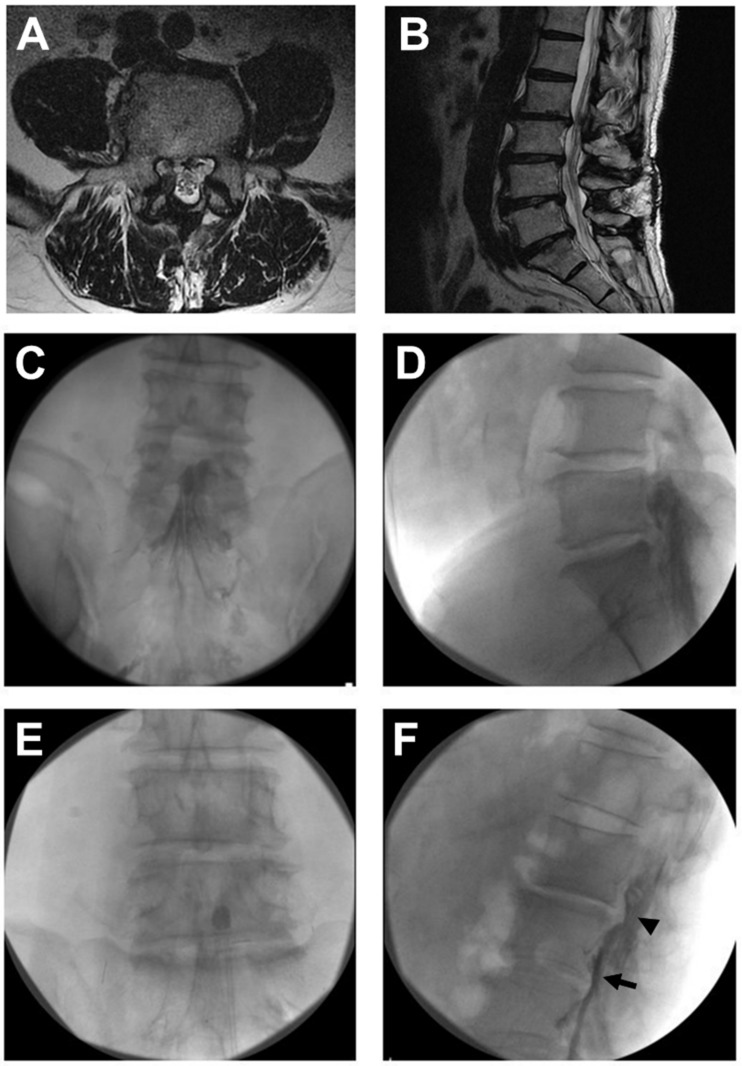
Percutaneous epidural adhesiolysis combined with balloon decompression in a patient with lumbar post-laminectomy syndrome. A 61-year-old male with a history of L4-5 partial laminectomy and flavectomy 1 year prior underwent combined epidural adhesiolysis and balloon decompression. (**A**) Adhesive cauda equina is shown in a cross-sectional magnetic resonance imaging (MRI) image at L4-5 level. (**B**) A sagittal MRI image of identical patient shows well-decompressed state in L4-5 level. (**C**) Anteroposterior fluoroscopic view showing a definite contrast filling defect in the previously operated L4-5 level. (**D**) A lateral fluoroscopic image showing a filling defect above the L4-5 level. (**E**) The inflatable balloon catheter placed in the suspected adhesion site and balloon filled with the contrast medium. (**F**) Contrast flow detected above the L4-5 level after the balloon procedure (arrowhead). The catheter is noted (arrow) in the anterior epidural space.

After sterile preparation before the procedure, a local anesthetic (lidocaine, 2%) was infiltrated under the skin and soft tissue. While waiting for sufficient local anesthesia, the balloon of the catheter (ZiNeu^®^, JUVENUI, Seoul, Korea; Figure 1) was prepared by filling a 1 mL Luer-Lock syringe (BD Medical, Franklin Lakes, NJ, USA) with 0.13 mL of contrast agent (Omnipaque, Nycomed Imaging AS, Oslo, Norway) before the procedure. A fluoroscopy C-arm system (OEC 9800, General Electric Healthcare, Little Chalfont, UK) was used for the procedure. A 10-gauge guide needle, specially designed for preventing various types of potential damage during catheter manipulation, was advanced through the sacral hiatus. The guide needle was gently introduced via the sacral hiatus under fluoroscopic image guidance. Consequently, approximately 8 mL of diluted contrast medium, which was prepared by mixing 4 mL of pure contrast medium (Omnipaque) and 4 mL of 1% lidocaine, was injected using the guide needle. If intravascular injection was detected, the needle was repositioned. After suitable identification via an epidurogram of the target areas, an inflatable balloon catheter was advanced through the guide needle to the filling defect sites or target areas as determined based on lumbar magnetic resonance imaging (MRI) findings and comprehensive assessment of symptoms before the procedure (Figure 2A,C,D). The target of the procedure was decided based on the symptoms and image findings. The epidural adhesiolysis and balloon decompression were performed via side-to-side positioning of the catheter with intermittent balloon inflating at the target lumbar level. For safety reasons, the ballooning was limited to 5 s each time (Figure 2E). Importantly, the balloon inflation time was adjusted based on the degree of pain caused by the procedure; if the patient complained of severe pain during balloon inflation, no further balloon decompression was attempted. Further, the catheter moved only when the balloon was deflated.

### 2.3. Outcome Assessments

Baseline characteristics, such as age, sex, body mass index, duration of pain, types of previous spine surgery, time points of previous spine surgery, neuropathic component, and pain intensity as measured using the NRS in the leg and back, were obtained for analysis. The evaluation of outcome was performed 1, 3, and 6 months after the procedure. All participants were evaluated using the following scales: an 11-point NRS from 0 (no pain) to 10 (worst possible pain) to determine the pain intensity of both leg and lower back; and the Korean version of the 10-item Oswestry Disability Index (ODI) questionnaire (range, 0–100; 0 = no disability) to determine the physical functional status. For analysis of the patient’s satisfaction and improvement after the procedure, the global perceived effect (GPE) after the procedure according to a 7-point Likert scale was also measured.

### 2.4. Definition of Treatment Response

According to previous studies, we defined the responder group with some modifications: (1) 50% (or ≥4-point) decrease of NRS score from baseline; (2) ≥30% (or ≥2-point) decrease of NRS score from baseline together with any one of the following criteria: (i) ≥30% (or ≥10-point) decrease in ODI from baseline or (ii) ≥5 points on the GPE scale [8,9,10]. Subjects who dropped out during the observation period were classified in the non-responder group regardless of the reason. Subjects who had any kinds of other additional interventional treatment or invasive treatment in the follow-up period were classified in the non-responder group.

### 2.5. Statistical Analysis

Continuous demographic data from the non-responders and responders were compared using the Student’s t-test or the Mann–Whitney U-test and are presented as means with standard deviations or medians with interquartile ranges as appropriate. Categorical demographic data were compared using a chi-square test or a Fisher’s exact test. Using univariate and multivariate regression analyses, the factors associated with a successful treatment response six months after combined epidural adhesiolysis and balloon decompression were analyzed. The most relevant factors associated with treatment response were included in the univariate logistic regression analysis. The inclusion of variables in the final multivariate logistic regression analysis to evaluate independent factors associated with treatment responses was based on biological plausibility, clinical importance, and statistical considerations. The quality of fit of the model was assessed with the Hosmer–Lemeshow test. A two-tailed *p*-value < 0.05 was considered to be statistically significant. The ability of pain duration after lumbar surgery to predict successful response after combined epidural adhesiolysis and balloon decompression was determined by calculating the area under the receiver operating characteristic (ROC) curve. The area under the ROC curve was calculated and the value with the highest sensitivity and specificity was set as the optimal cut-off value. The data were analyzed using the Statistical Package for the Social Sciences Version 21.0 (SPSS Inc., Chicago, IL, USA) and MedCalc version 11.3.3 (MedCalc Software, Mariakerke, Belgium).

## 3. Results

**Figure 3:**
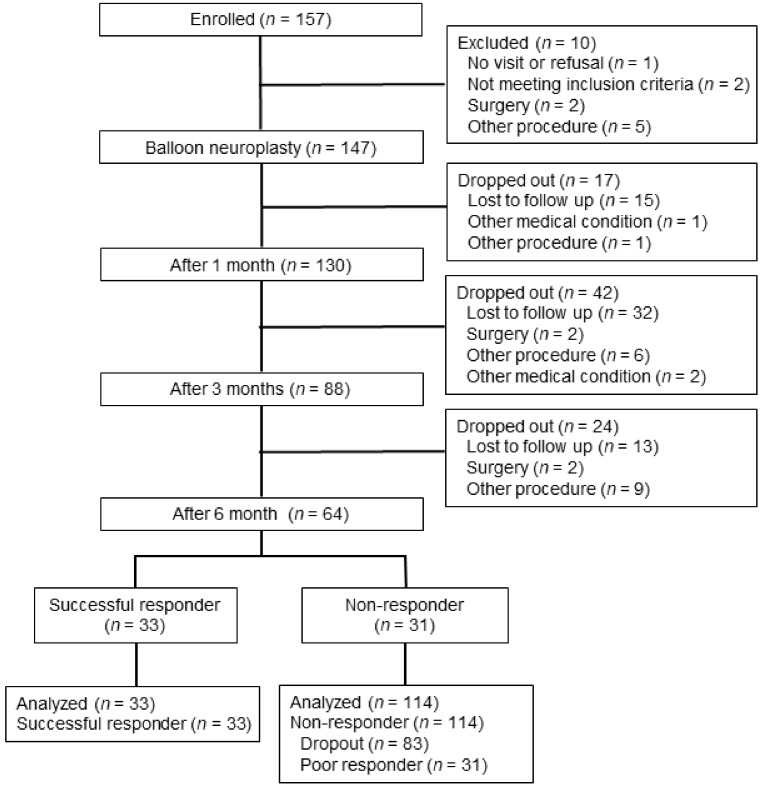
Schematic flow diagram depicting participant enrolment.

Figure 3 illustrates the participant selection. As a result of searching the ITSM, we initially found 157 subjects. Among these patients, 147 subjects finally met the inclusion criteria. During follow-up, 1, 3, and 6 months after the balloon procedure, 17, 42, and 24 subjects dropped out, respectively. At the six-month follow up, 33 subjects were classified into the responder group and 114 subjects were classified into the non-responder group based on a robust combination of outcome measures described above.

The overall baseline demographic characteristics of the 147 patients are shown in Table 1. We analyzed outcomes at 1, 3, and 6 months and the proportion of responders at each follow-up time-point is summarized in Table 2. The percentages of patients who exhibited successful treatment responses were 32.0%, 24.5%, and 22.4% of the study population at 1, 3, and 6 months, respectively (Table 2). The demographic characteristics of non-responders and responders at six months after the procedure are shown in Table 3. Upon comparison of the demographic characteristics between the two groups at six months after balloon adhesiolysis, no significant differences were noted except that the non-responders had a longer duration of pain symptoms than the responders (*p* = 0.002, Table 3). The intervention characteristics were also not significantly different between the two groups (Table 4).

**Figure 4:**
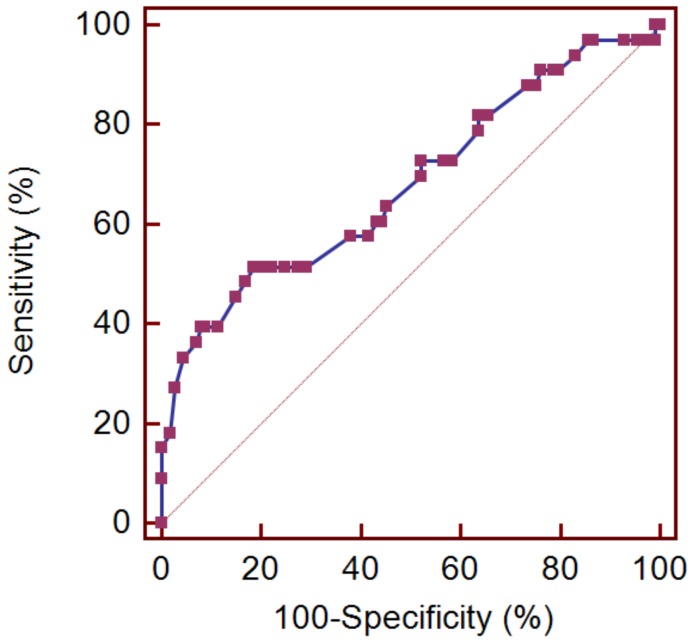
Receiver operating characteristic curve analysis to determine the ability of pain duration after lumbar surgery to predict successful response to combined epidural adhesiolysis and balloon decompression. The best cut-off point was ≤14 months, with a sensitivity of 51.5%, a specificity of 81.4%, and an AUC of 0.680 (*p* = 0.002).

Univariate logistic regression analysis showed that the duration of pain after lumbar spine surgery was a significant factor associated with successful outcome at six months after the balloon procedure (odd ratio (OR) = 0.986, 95% confidence interval (CI) = 0.972–0.999, *p* = 0.042). In addition, according to our previous study [8], possible predictive factors, such as age, diabetes, and spondylolisthesis, were included in the univariate logistic regression analysis. However, factors such as age, number of previous lumbar surgeries, and presence of diabetes and spondylolisthesis were not found to be factors associated with the six-month outcome of balloon adhesiolysis. Considering a meaningful *p*-value for statistical difference in the comparison of patient characteristics to be 0.2, the number of target lumbar levels was included in the multivariate logistic regression analysis. After adjusting for demographic differences, clinical importance, and biologic plausibility in the multivariate regression analysis, only pain duration was independently associated with a successful outcome at six months after balloon adhesiolysis (OR = 0.985, 95% CI = 0.971–0.999; *p* = 0.038, Table 5). In addition, the ROC curve analysis showed that the area under the curve of pain duration after lumbar surgery was 0.680 (95% CI = 0.597–0.754, *p* = 0.002), with an optimal cut-off value of ≤14 months, sensitivity of 51.5%, and specificity of 81.4% (Figure 4).

The complications observed during balloon adhesiolysis are shown in Table 6. In addition, some patients reported residual pain in the post-procedural period. However, temporary pain aggravation was alleviated spontaneously within two to three days. Although there were some possible complications, such as dural puncture or suspected vascular administration of drugs, no additional medications or treatments were required. Notably, one patient showed transient motor weakness after the balloon procedure that improved spontaneously during the follow-up period. None of the patients experiencing these complications had any persistent neurologic abnormalities. All patients were discharged after bed rest for a short time after the procedure.

## 4. Discussion

To date, diagnosing PLS remains a controversial issue [12]. The concept of PLS was first described by North et al. in 1991, termed as failed back surgery syndrome [13]. However, the diagnostic criteria for PLS remain uncertain, with some authors asserting that it is a misnomer [5,12,14]. In this study, we considered lumbar PLS to be characterized by ongoing pain symptoms of at least three months duration post completion of a surgical procedure according to the most recently proposed criteria [11]. The symptoms of lumbar PLS can be induced by post-surgical fibrotic scar or adhesional fibrosis, remnant disc fragment, adjacent spondylosis at the fusion site, underlying hardware pain, or even inexplicable neuropathic pain [5,15,16]. Therefore, management of lumbar PLS might require a multidimensional approach [17], including epidural adhesiolysis. Previous studies showed that percutaneous epidural adhesiolysis without balloon procedure in patients with lumbar PLS may be a beneficial treatment option [18,19]. We found the success rate in this study to be relatively limited in contrast to that in our previous study in patients without lumbar surgery [8,9,10]. Indeed, endoscopic adhesiolysis was shown to be more effective for lumbar PLS in the short term (<6 months) than in the long term (>6 months) [20]. Specifically, the ODI values were restored to baseline in most of the patients at the one-year follow-up in another study [21]. Similarly, the present results showed that the number of responders at the one- and six-month follow-up was 47 (32.0%) and 33 (22.4%), respectively (Figure 2). It seemed that if a successful response was observed after combined epidural adhesiolysis and balloon decompression in patients with lumbar PLS, 70.2% of these patients with treatment response could maintain the effect for at least six months. In this context, our results might be comparable to those of a previous study [20]. Therefore, determining the predictive factors of response after combined epidural adhesiolysis and balloon decompression in patients with lumbar PLS is important. In addition, cost-effectiveness is another issue with regards to epidural adhesiolysis in patients with lumbar PLS. For example, Brito-García pointed out the sub-par cost-effectiveness of adhesiolysis for lumbar PLS [22]. Although a direct comparison is not appropriate, we cautiously presume that combined epidural adhesiolysis and balloon decompression could be an effective alternative treatment option in terms of cost-effectiveness. However, further cost-effectiveness studies will be required with regards to the combined epidural adhesiolysis and balloon decompression treatment approach for lumbar PLS.

As mentioned above, determination of the predictive factors of response after the balloon procedure in lumbar PLS is important. Multivariate regression analysis in this study showed that a short duration of pain after lumbar surgery may be associated with a favorable outcome after combined epidural adhesiolysis and balloon decompression. Moreover, the present ROC curve analysis found that an optimal cut-off value was ≤14 months of pain duration after lumbar surgery to predict the successful response to combined epidural adhesiolysis and balloon decompression. This result could be explained based on the duration of post-operative adhesion formation. Presumably, an old fibrotic adhesional scar is harder than a recent one. The degree of fibrotic adhesion is directly proportional to the effectiveness of adhesiolysis and drug administration at the targeted sites. Additionally, when compared to our previous study [8], the analysis in the present study showed that the presence of diabetes was not associated with the outcomes of the balloon procedure in patients with lumbar PLS. As pain symptoms of PLS often manifest with neuropathic components as in diabetes [16], it may be difficult to determine the real association between diabetes and the outcomes of balloon adhesiolysis.

Our previous study showed the effectiveness of combined epidural adhesiolysis and balloon decompression in patients with chronic intractable lumbar radicular pain without previous lumbar surgery [8,9,23]. Despite the relatively unsatisfactory outcomes, the present results corroborate those published in the previous report considering the poor response to conventional treatment approaches [24]. Therefore, combined epidural adhesiolysis and balloon decompression treatment could be an alternative in cases of lumbar PLS with poor response to conventional treatment.

With regards to complications, one patient with lumbar PLS experienced temporary motor weakness after balloon decompression. Even though this motor weakness spontaneously resolved, interventionists should consider the possibility of this neurologic deficit after the procedure, especially in patients with lumbar PLS. The occurrence of this complication may be attributable to transient ischemia during ballooning. Therefore, the balloon should be gently inflated while observing the patient’s response. If the patient complains of severe pain during balloon inflation, no further balloon decompression should be attempted. In addition, the rate of dura puncture during the balloon procedure in patients with lumbar PLS is two times that in patients without previous lumbar surgery [9,10]. The reason for this difference is presumed to be post-operative adhesion. Inadvertent dura puncture during adhesiolysis might not lead to a catastrophic complication; however, it makes further adhesiolysis difficult because of possible intrathecal injection. Thus, patients should be monitored carefully for dura puncture during the procedure, especially in lumbar PLS cases.

This study had some limitations. Firstly, we did not include medication use in the analysis of the outcomes. In most cases, patients were taking sufficient amounts of pain killers including opioids, the use of which may lead to meaningful variations in the outcome [25]. To overcome this limitation, we evaluated the GPE, which is considered to take into account medication use. Secondly, the definition of treatment response used in this study differed slightly from that used in previous studies, and a different definition may have led to different results [8]. Thirdly, we classified patients who were lost to follow-up in the non-responder group. However, this classification might have been underestimated with our strict analysis, since patients with a follow-up loss despite the successful response would be classified as a non-responder. Fourthly, we did not consider electrophysiologic studies for diagnosing lumbar PLS following the current definition [11]. Electrodiagnostic testing, such as electromyography and nerve conduction velocities, can help differentiate between radiculopathy and other peripheral neuropathic conditions [26]. However, electrodiagnosis has limitations for patients with laminectomy which may give paraspinal muscle false positives [27]. Nevertheless, we did not exclude that it may affect the results of the present study. Lastly, this study was retrospective in nature, with no control or sham group to conduct an accurate comparison of the outcomes of the procedure. A randomized controlled trial may be needed to address this limitation.

## 5. Conclusions

The combined epidural adhesiolysis and balloon decompression approach may have limited effectiveness in patients with lumbar PLS. However, early intervention with combined epidural adhesiolysis and balloon decompression after development of lumbar PLS may be associated with favorable outcomes.

## Figures and Tables

**Table 1:** Baseline characteristics of the study subjects.

Parameters	*n* = 147
Age (years)	68.0 (60.0–74.0)
Sex (male/female)	58 (39.5%)/89 (60.5%)
Height (cm)	159.0 (152.5–167.0)
Weight (kg)	64.0 (57.0–70.5)
Body mass index (kg/m^2^)	25.0 ± 3.1
Duration of pain (months)	36.0 (14.0–60.0)
Types of previous spine surgery	
Discectomy and laminectomy	72 (49.0%)
Fusion surgery	68 (46.3%)
Others	7 (4.8%)
Number of previous spine surgery	
1/2/3 or more	114 (77.6%)/24 (16.3%)/9 (6.1%)
Symptom	
Lower back pain/leg pain/both	33 (22.4%)/33 (22.4%)/81 (55.1%)
Concurrent disease	
Diabetes/Hypertension	36 (24.5%)/69 (46.9%)
Neuropathic component	60 (40.8%)
Previous neuroplasty	35 (23.8%)
Spondylolisthesis	72 (49.0%)

Data are expressed as numbers (%) and means ± standard deviation or medians (interquartile range).

**Table 2:** Proportion of responders to treatment after combined epidural adhesiolysis and balloon decompression in patients with lumbar post-laminectomy syndrome.

	Follow-Up	Number (%)
	(Months)	*n* = 147
Successful responders	1	47 (32.0%)
	3	36 (24.5%)
	6	33 (22.4%)

Successful response after the procedure was defined as: (1) a decrease of ≥50% or ≥4 points on the numerical rating scale; or (2) a decrease of ≥30% or ≥2 points on the numerical rating scale, including one of the following conditions: (i) decrease of ≥30% or ≥10 points in ODI, or (ii) ≥5 points on the GPE scale. ODI = Oswestry Disability Index; GPE = global perceived effect.

**Table 3:** Characteristics of the non-responders and responders at six months after combined epidural adhesiolysis and balloon decompression in patients with lumbar post-laminectomy syndrome.

	Non-Responder	Successful Responder	*p*-Value
	(*n* = 114)	(*n* = 33)	
Age (years)	68.0 (60.0–75.0)	67.0 (60.5–71.0)	0.257
Sex (male/female)	48 (40.7%)/70 (59.3%)	13 (39.4%)/20 (60.6%)	0.894
Weight	64.0 (56.2–70.0)	64.1 (57.0–71.3)	0.679
Height (cm)	158.0 (152.0–168.0)	160.0 (153.5–166.7)	0.673
BMI (kg/m^2^)	25.0 ± 3.2	25.1 ± 2.9	0.924
Duration of pain	36.0 (19.0–67.0)	14.0 (6.0–48.0)	0.002
BDI	16.7 ± 12.3	14.0 ± 8.4	0.690
ODI	48.6 ± 19.0	50.0 ± 16.0	0.769
Pain intensity (NRS)			
Lower back pain	7.3 ± 1.6	7.5 ± 1.9	0.605
Leg pain	7.3 ± 1.6	7.2 ± 1.6	0.692
Previous surgery type			0.678
Discectomy	58 (50.9%)	14 (42.4%)	
Fusion surgery	51 (44.7%)	17 (51.5%)	
Others	5 (4.4%)	2 (6.1%)	
Previous surgery number			0.161
1	85 (74.6%)	29 (87.9%)	
2	20 (17.5%)	4 (12.1%)	
3 or more	9 (7.9%)	0 (0.0%)	
Symptom			0.449
Lower back pain	28 (24.6%)	5 (15.2%)	
Leg pain	26 (22.8%)	7 (21.2%)	
Both	60 (52.6%)	21 (63.6%)	
Diabetes	28 (24.6%)	8 (24.2%)	0.970
Hypertension	51 (44.7%)	18 (54.5%)	0.320
Neuropathic component	48 (42.1%)	12 (36.4%)	0.555
Previous neuroplasty	28 (24.6%)	7 (21.2%)	0.691
Spondylolisthesis	55 (48.2%)	17 (51.5%)	0.741

Data are expressed as numbers (%) and means ± standard deviation. BMI = body mass index; BDI = Beck depression inventory; ODI = Oswestry Disability Index; NRS = numeric rating scale.

**Table 4:** Intervention characteristics of the non-responders and the successful responders at six months after combined epidural adhesiolysis and balloon decompression in patients with lumbar post-laminectomy syndrome.

	Non-Responder	Successful Responder	*p*-Value
	(*n* = 114)	(*n* = 33)	
Treatment location			0.387
Left/Right	21 (18.4%)/15 (13.2%)	8 (24.2%)/6 (18.2%)	
Both	15 (13.2%)	4 (12.1%)	
Central only	18 (15.8%)	5 (15.2%)	
Central with unilateral foramen (left/right)	11 (9.6%)/8 (7.0%)	6 (18.2%)/2 (6.1%)	
Central with both foramina	26 (22.8%)	2 (6.1%)	
Target level			0.068
One	53 (46.5%)	13 (39.4%)	
Two	44 (38.6%)	19 (57.6%)	
Three and more	17 (14.9%)	1 (3.0%)	

Data are expressed as numbers (%).

**Table 5:** Logistic regression analysis of factors associated with treatment response at six months after combined epidural adhesiolysis and balloon decompression in patients with lumbar post-laminectomy syndrome.

Parameters	Univariate			Multivariate		
	OR	95% CI	*p*-Value	OR	95% CI	*p*-Value
Age	0.981	0.94–1.014	0.253			
Duration of pain	0.986	0.972–0.999	0.042	0.985	0.971–0.999	0.038
Number of surgeries						
One (reference)	1					
Two	0.586	0.185–1.857	0.364			
Three or more	0.000	0.000	0.999			
Diabetes						
No (reference)	1					
Yes	0.983	0.398–2.425	0.970			
Spondylolisthesis						
No (reference)	1					
Yes	1.140	0.525–2.475	0.741			
Target level						
One (reference)	1			1		
Two	1.760	0.783–3.961	0.172	1.945	0.845–4.478	0.118
Three and more	0.240	0.029–1.970	0.184	0.273	0.033–2.279	0.231

OR = odds ratio; CI = confidence interval.

**Table 6:** Complications observed after combined epidural adhesiolysis and balloon decompression in patients with lumbar post-laminectomy syndrome.

Complication	Number (%)
Suspected dura puncture	13 (8.8)
Temporary motor weakness	1 (0.6)
Vascular injection	1 (0.6)
Disc injection	0 (0.0)
Coccydynia	1 (0.6)
